# Supplementary material for: Multi‐dataset identification of innovative feature genes and molecular mechanisms in keratoconus
Source: J Cell Mol Med. 2024 Sep 19;28(18):e70079. doi: 10.1111/jcmm.70079 (PMC11412914; doi:10.1111/jcmm.70079)
Supplement: Supplementary file 2 — Material S2. [file JCMM-28-e70079-s002.zip › 7.GSEA analysis/6.SNORA74B/KEGG_ASTHMA.html]

Details for gene set KEGG\_ASTHMA[GSEA]

|  || Dataset | SNORA74B.group.cls#h\_versus\_l |
| Phenotype | group.cls#h\_versus\_l |
| Upregulated in class | l |
| GeneSet | KEGG\_ASTHMA |
| Enrichment Score (ES) | -0.6294348 |
| Normalized Enrichment Score (NES) | -1.3928494 |
| Nominal p-value | 0.09142857 |
| FDR q-value | 0.34072036 |
| FWER p-Value | 0.908 |
Table: GSEA Results Summary

  

Fig 1: Enrichment plot: KEGG\_ASTHMA      
 Profile of the Running ES Score & Positions of GeneSet Members on the Rank Ordered List

  

| SYMBOL | TITLE | RANK IN GENE LIST | RANK METRIC SCORE | RUNNING ES | CORE ENRICHMENT || 1 | IL9 | na | 2712 | 0.319 | -0.0302 | No |
| 2 | IL3 | na | 5142 | 0.221 | -0.0669 | No |
| 3 | IL13 | na | 9160 | 0.110 | -0.1662 | No |
| 4 | HLA-DRB5 | na | 11281 | 0.070 | -0.2167 | No |
| 5 | HLA-DQB1 | na | 13160 | 0.045 | -0.2640 | No |
| 6 | CCL11 | na | 13754 | 0.039 | -0.2753 | No |
| 7 | EPX | na | 14071 | 0.035 | -0.2791 | No |
| 8 | HLA-DOA | na | 16250 | 0.012 | -0.3401 | No |
| 9 | HLA-DRB1 | na | 19037 | -0.017 | -0.4178 | No |
| 10 | HLA-DMA | na | 19878 | -0.027 | -0.4379 | No |
| 11 | FCER1G | na | 22025 | -0.052 | -0.4919 | No |
| 12 | IL5 | na | 23454 | -0.072 | -0.5222 | No |
| 13 | HLA-DOB | na | 23582 | -0.074 | -0.5147 | No |
| 14 | HLA-DRB4 | na | 24359 | -0.087 | -0.5240 | No |
| 15 | HLA-DRB3 | na | 24377 | -0.087 | -0.5114 | No |
| 16 | PRG2 | na | 28188 | -0.170 | -0.5957 | Yes |
| 17 | IL10 | na | 28370 | -0.176 | -0.5745 | Yes |
| 18 | CD40LG | na | 30278 | -0.236 | -0.5940 | Yes |
| 19 | HLA-DRA | na | 30472 | -0.244 | -0.5628 | Yes |
| 20 | HLA-DPA1 | na | 30909 | -0.263 | -0.5357 | Yes |
| 21 | MS4A2 | na | 31010 | -0.268 | -0.4982 | Yes |
| 22 | TNF | na | 31738 | -0.308 | -0.4727 | Yes |
| 23 | HLA-DMB | na | 32287 | -0.339 | -0.4375 | Yes |
| 24 | CD40 | na | 32420 | -0.349 | -0.3888 | Yes |
| 25 | IL4 | na | 32876 | -0.383 | -0.3442 | Yes |
| 26 | FCER1A | na | 33985 | -0.529 | -0.2965 | Yes |
| 27 | HLA-DPB1 | na | 34158 | -0.570 | -0.2157 | Yes |
| 28 | HLA-DQA2 | na | 34566 | -0.745 | -0.1154 | Yes |
| 29 | HLA-DQA1 | na | 34604 | -0.798 | 0.0036 | Yes |
Table: GSEA details [plain text format]

  

Fig 2: KEGG\_ASTHMA      
 Blue-Pink O' Gram in the Space of the Analyzed GeneSet

  

Fig 3: KEGG\_ASTHMA: Random ES distribution      
 Gene set null distribution of ES for **KEGG\_ASTHMA**
